# Supplementary figures and images for: The neuroendocrine transition in prostate cancer is dynamic and dependent on ASCL1
Source: bioRxiv. 2024 Apr 11:2024.04.09.588557. Preprint. [Version 2] doi: 10.1101/2024.04.09.588557 (PMC11030418; doi:10.1101/2024.04.09.588557)

Source Data Fig 1:

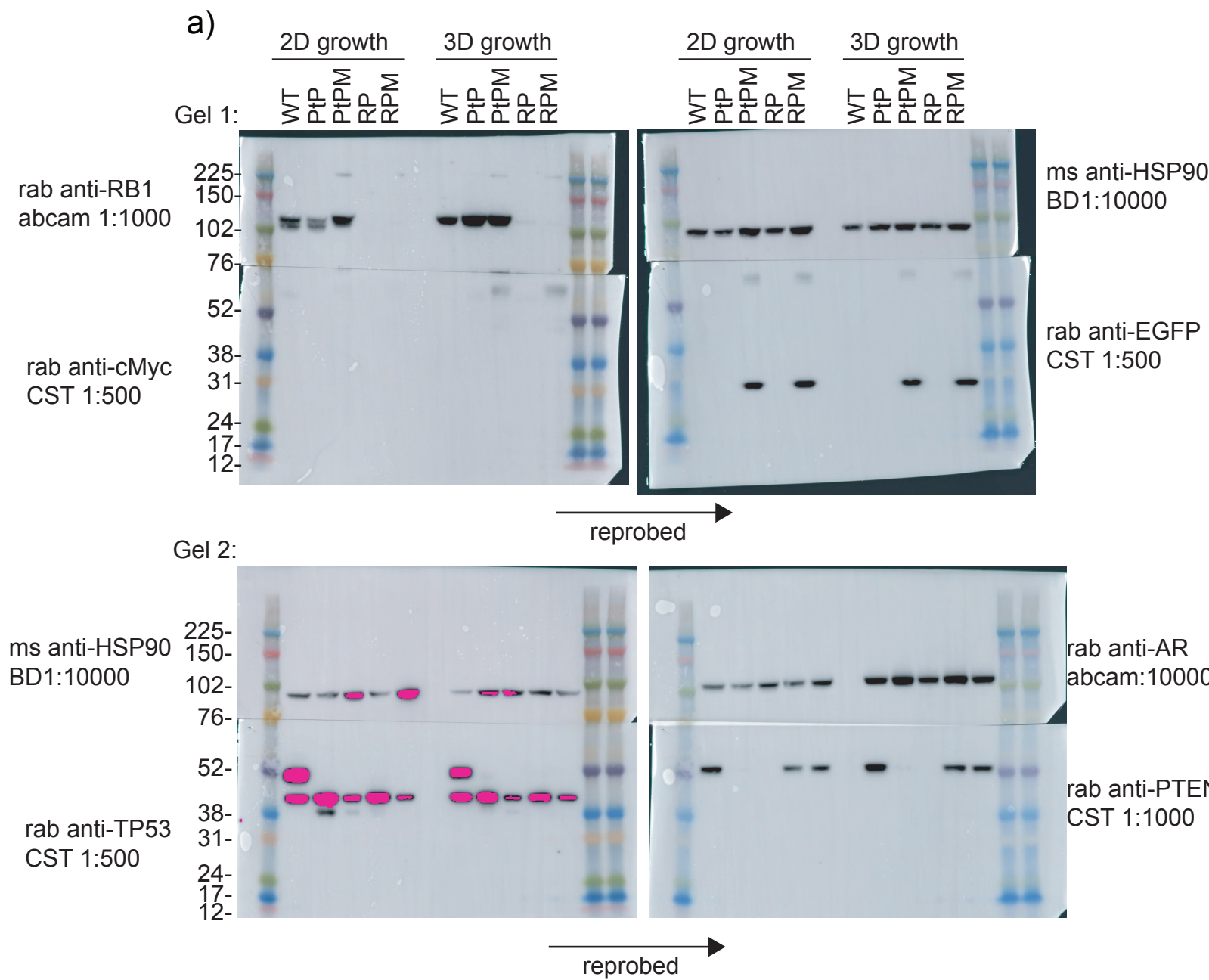

Supplement: Supplement 4 — Source Data Figure 1: Unedited western blot scans (related to Extended Data Fig. 2c). [file media-4.pdf]
